# Supplementary material for: The Coronavirus Health and Impact Survey (CRISIS) reveals reproducible correlates of pandemic-related mood states across the Atlantic
Source: medRxiv. 2020 Aug 27:2020.08.24.20181123. Preprint. [Version 1] doi: 10.1101/2020.08.24.20181123 (PMC7457620; doi:10.1101/2020.08.24.20181123)
Supplement: 1 [file NIHPP2020.08.24.20181123-supplement-1.pdf]

medRxiv preprint doi: https://doi.org/10.1101/2020.08.24.20181123.this version posted August 27, 2020. The copyright holder for this preprint (which was not certified by peer review) is the author/funder, who has granted medRxiv a license to display the preprint in perpetuity.
It is made available under a CC-BY 4.0 International license .

doi:10.1101/2020.05.19.20106344.

48. Alexander, L. M. *et al.* An open resource for transdiagnostic research in pediatric mental health and learning disorders. *Sci Data* **4**, 170181 (2017).

49. Tumer, K. & Ghosh, J. Linear and order statistics combiners for pattern classification. CoRR. *arXiv preprint cs. NE/9905012* (1999).

50. Li, H. *et al.* K-Means Clustering with Bagging and MapReduce. in *2011 44th Hawaii International Conference on System Sciences* 1–8 (ieeexplore.ieee.org, 2011).

51. Friedman, J., Hastie, T. & Tibshirani, R. *The elements of statistical learning*. vol. 1 (Springer series in statistics New York, 2001).

52. Brandes, U. *et al.* On Modularity Clustering. *IEEE Trans. Knowl. Data Eng.* **20**, 172–188 (2008).

53. Strobl, C., Boulesteix, A.-L., Zeileis, A. & Hothorn, T. Bias in random forest variable importance measures: illustrations, sources and a solution. *BMC Bioinformatics* **8**, 25 (2007).

Supplemental Materials:

Supplemental Methods

Overview: First, we used confirmatory factor analysis to assess the structure of each of the domains and inform the calculation of factor scores for unidimensional constructs. Test-retest reliability was calculated for unidimensional factors and individual items using ICC(3,1).[30] We used Louvain community detection, a clustering technique, to meaningfully summarize domains for which unidimensional factors exhibited poor fit. Finally, we used random forests to demonstrate the construct validity of the CRISIS by assessing the importance of the included domains in predicting the Current Mood States factor.

Subtyping: We use bagging-enhanced Louvain Community Detection to discover groups of individuals that have profiles across both the Life Changes questions, and the Daily Behaviors, Media Use, and Substance Use questions, which we called the Prior Habits subtypes. Louvain Community Detection is known to robustly link observations together through the use of an iterative modularity-optimizing procedure to find groups of individuals. Other clustering approaches, such as K-means, or spectral clustering, require the experimenter to

medRxiv preprint doi: https://doi.org/10.1101/2020.08.24.20181123.this version posted August 27, 2020. The copyright holder for this preprint
(which was not certified by peer review) is the author/funder, who has granted medRxiv a license to display the preprint in perpetuity.
It is made available under a CC-BY 4.0 International license .

choose the resolution of the clustering a priori, which can be problematic and lead to instability across samples.[49–51] Louvain Community detection on the other hand, through iterative permutations of individuals in each community, optimizes for modularity, a commonly used metric of cluster quality.[32,52] We enhance the reproducibility of our subtyping method through the use of bootstrap aggregation, or bagging. Using bootstrap aggregated clustering creates more reproducible clusters by reducing variability that may occur due to random variations in sample composition.

Random Forest: Briefly, the RF algorithm creates a series of decision trees for which a random selection of variables are chosen and a bootstrapped sample is used to train the model. For each iteration of the 1000 bootstrap runs, the performance on each of these decision trees on the out-of-sample data, roughly ⅓ of the sample, is aggregated and used to assess the performance of the RF model. (For a review of RF, see[51]). RF provides a robust assessment of the relative impact of each of these variables in predicting outcomes, known as variable importance, which we assess for each variable in our predictive model. To protect against overfitting, we create a null performance distribution from our own data by shuffling the outcome variable and repeating the random forest prediction pipeline 1000 times. The out-of-sample prediction R-squared value is then calculated for our prediction and compared to the distribution of these 1000 shuffled null models. We assess if our predictive accuracy surpasses the 99.9999% confidence interval of the null model. We used COVID Worries and Prior Mood States transformed into quintiles in order to protect against the inflation effects that RF can have on the variable importance of continuous versus categorical variables.[53]

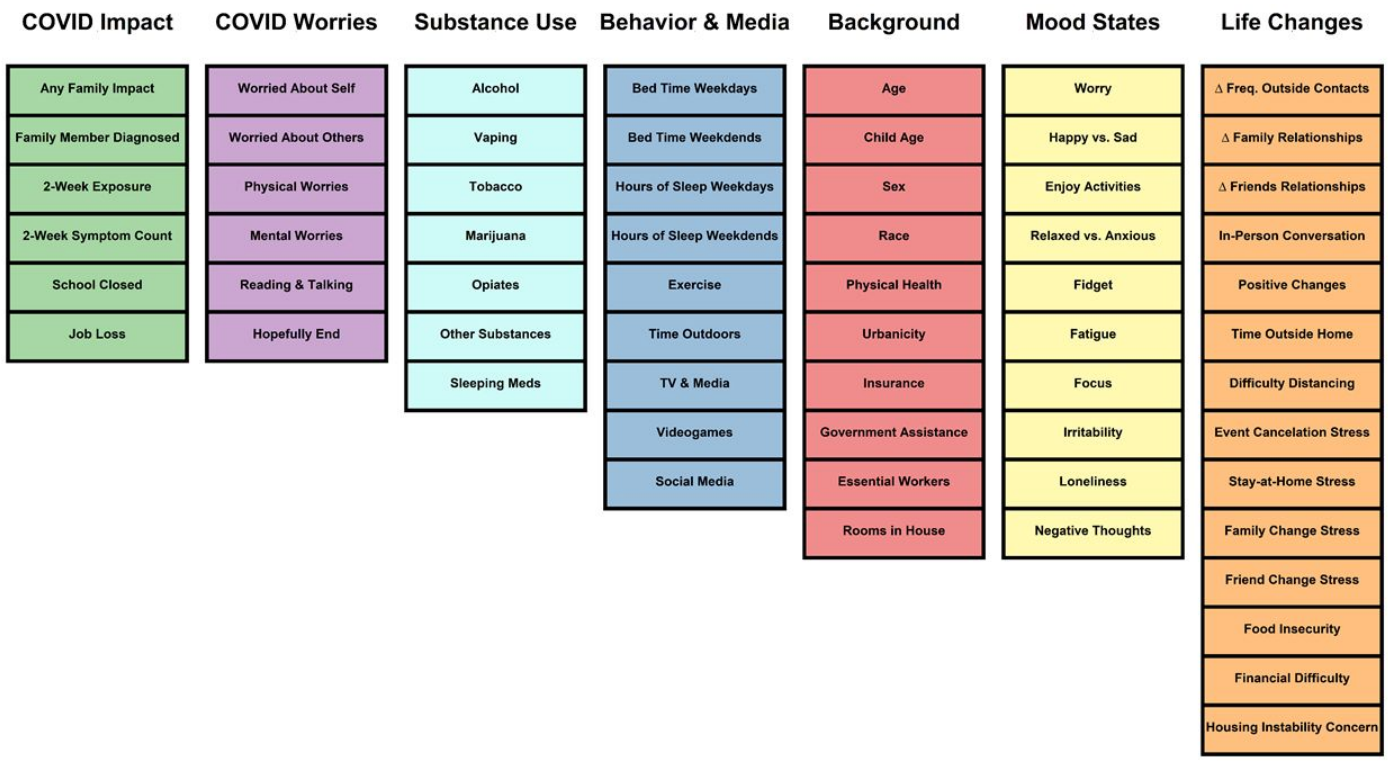

S. Figure 1: Columns contain the variables included in each of the individual categories and colored by category respectively.

medRxiv preprint doi: https://doi.org/10.1101/2020.08.24.20181123.this version posted August 27, 2020. The copyright holder for this preprint (which was not certified by peer review) is the author/funder, who has granted medRxiv a license to display the preprint in perpetuity.
It is made available under a CC-BY 4.0 International license .

Supplemental Results

Prior Habits Subtyping: We found 3 Prior Habits subtypes in both US and UK adult samples. Individuals in subtype 1 spend the least amount of time exercising and outdoors, and in the US go to sleep relatively later and spend more time on media, while in the UK this subtype goes to sleep earlier and spends an average amount of time on media. Subtype two goes to bed later, in the US this subtype gets relatively less sleep but spends more time exercising and outdoors, while in the UK this group gets an average amount of sleep and time spent exercising and outdoors. Subtype 3 goes to bed earliest and spends more time exercising and outdoors, and reports relatively lower media use and drug use.

Across US and UK samples these subtypes were highly reproducible, with high Pearson's correlations between subtype mean scores (r 0.71 – 0.96) for the adult sample. ANOVA revealed Prior Habits subtypes had different Prior Mood States factor (US $p< 0.00001$, UK $p< 0.00001$) but ANCOVA of Current Mood States controlling for Prior Mood states only differed in the US sample (US $p > 0.05$, UK $p > 0.05$). The US sample also showed different Mood States scores by subtype (US $p < 0.00001$; UK $p = 0.06$). COVID Worries scores differed by subtype in the UK but not US (US $p > 0.05$, UK $p < 0.01$). Mean factor scores by subtype are shown in the Supplemental table 3. Overall, results indicated significant differences between subtypes in prior mood states scores in both US and UK, with subtype 1 showing highest scores in the US and subtype 3 showing highest scores in the UK. We also see significant subtype differences by age in both US and UK. Prior Habits subtypes also differed by key demographic variables including age, race, education, rooms in house, household density, and employment status (see Supplement).

The parent report US and UK subtypes were highly reproducible, with high Pearson's correlations between subtype mean scores (r 0.97 – 0.99) In the parent report data we found 3 Prior Habit subtypes in the US sample and 4 in the UK sample. Individuals in subtype 1, in both the US and UK went to bed later in the evening and got the least amount of sleep, and also spent less time exercising and outdoors. Subtype 1 also reports the highest media use and ratings of drug use. Subtype 2 went to bed early and got above average sleep, but below average exercise, outdoor time, and social media use. Compared to previous subtypes, Subtype 3 showed relatively divergent patterns across the US and UK, with the US sample showing later than average bedtime and media use, less than average sleep, while in the UK individuals had an early bedtime but greater than average sleep and less media use on average. Subtype 4, in the UK only, showed the earliest bedtime, and the greatest amount of sleep, exercise and outdoor time, and the least amount of media use.

One-way ANOVA of subtype by Prior Mood score and ANCOVA of Current Mood factor score, controlling for Prior Mood Score, indicates that these Prior Habits subtypes show different patterns of Mood States over time (Prior Mood State: US $p > 0.05$, UK $p< 0.00001$; Current Mood State: US $p > 0.05$, UK $p > 0.05$). One-way ANOVA of subtype by COVID Worries factor score shows that these Prior Habits subtypes are sensitive to differences in COVID worries in the UK but not US (US $p < 0.05$, UK $p < 0.00001$). The parent report subtypes show subtype 1 and 2 with the highest and lowest COVID worries factor scores respectively. We also see that subtype 1 and 2 are significantly different in age distribution, with subtype 1 having the most teenagers and subtype 2 having the most children under 5.

medRxiv preprint doi: https://doi.org/10.1101/2020.08.24.20181123.this version posted August 27, 2020. The copyright holder for this preprint
(which was not certified by peer review) is the author/funder, who has granted medRxiv a license to display the preprint in perpetuity.
It is made available under a CC-BY 4.0 International license .

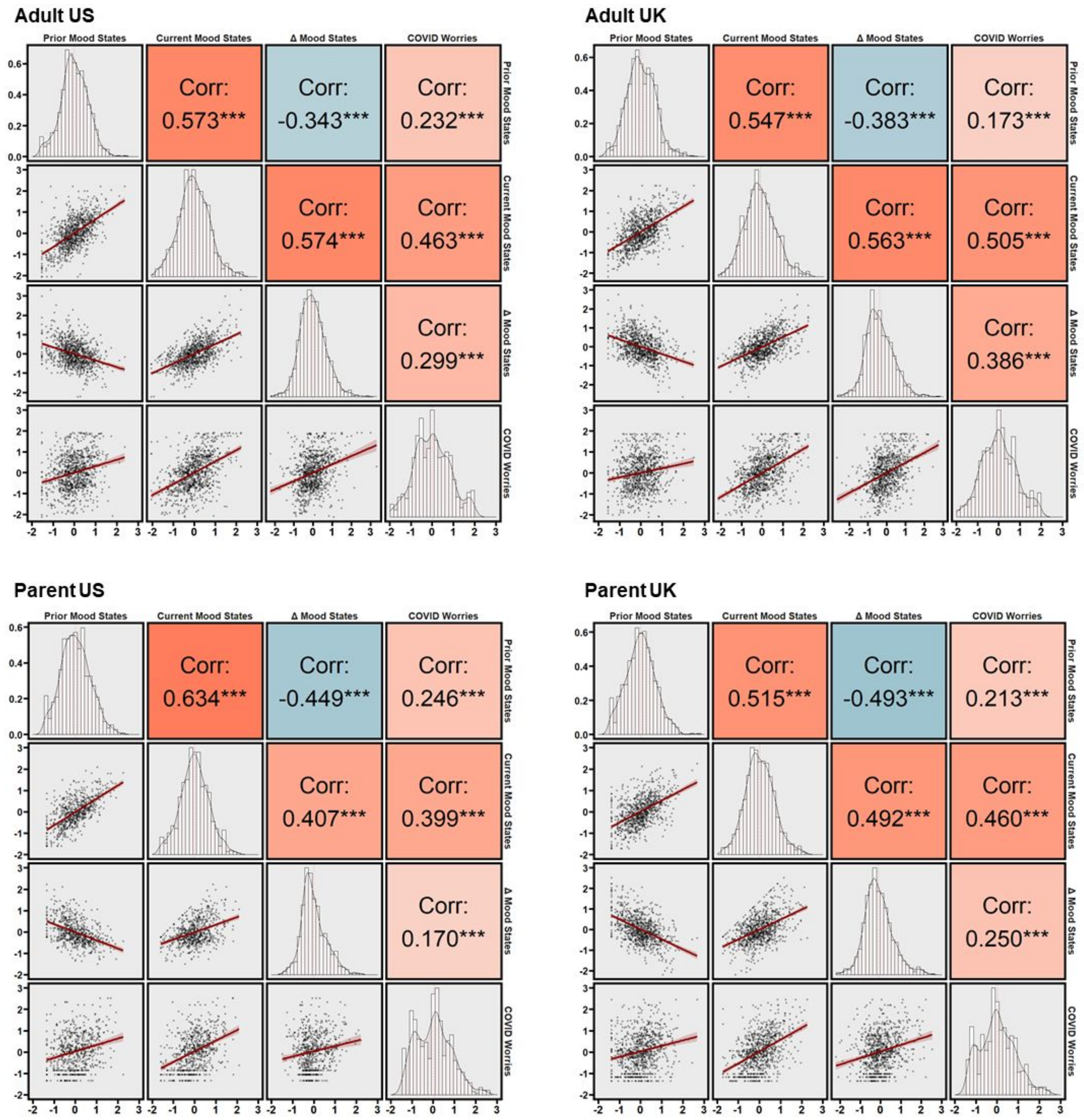

S Figure 2. Correlations between all factors scores (Prior Mood States, Current Mood States, Δ Mood States, and COVID Worries) across all samples. Lower left panels display matrices of scatter plots of the correlation between the factors. X and Y label values represent a standard loading of -2 at the origin and 3 at the maximum for each of the factors. Corresponding diagonal panels show the histogram distribution of the factor correlations. Pearson correlation coefficient values are presented in the top right panels.

medRxiv preprint doi: https://doi.org/10.1101/2020.08.24.20181123.this version posted August 27, 2020. The copyright holder for this preprint (which was not certified by peer review) is the author/funder, who has granted medRxiv a license to display the preprint in perpetuity.
It is made available under a CC-BY 4.0 International license .

	US Adult	UK Adult	US Parent	UK Parent
**Missing Data**				
Mean (SD)	0.6 (1.8)	0.5 (1.1)	0.4 (0.9)	0.4 (1.0)
**Range of Missed Items**	0-54	0-17	0-14	0-12
**Time to Completion**				
Mean (SD)	13.9 (13.1)	14.4 (11.5)	14.1 (7.5)	13.9 (20.9)
**Incomplete Observations**				
N (%)	108 (6.0)	43 (2.6)	43 (3.5)	88 (5.4)

S. Table 1. We summarize missing data here. First, the number of items missing on average in each completed survey; Second, the range of missing items across all completed surveys, and third the average time to completion of the surveys (in minutes). Fourth, the total number of incomplete surveys for each sample.

medRxiv preprint doi: https://doi.org/10.1101/2020.08.24.20181123.this version posted August 27, 2020. The copyright holder for this preprint (which was not certified by peer review) is the author/funder, who has granted medRxiv a license to display the preprint in perpetuity.
It is made available under a CC-BY 4.0 International license .

	Parent US Mean (SD)			Parent UK Mean (SD)		
	Covid Worries	Prior Mood States	Current Mood States	Covid Worries	Prior Mood States	Current Mood States
**Population**	0.04 (0.83)	0.01 (0.69)	0.004 (0.67)	0.03 (0.83)	0.007 (0.69)	0.004 (0.68)
**Sex**						
Male	0.03 (0.85)	0.05 (0.67)	0.01 (0.69)	0.02 (0.84)	0.03 (0.69)	0.04 (0.67)
Female	0.05 (0.81)	-0.04 (0.70)	-0.002 (0.64)	0.03 (0.81)	-0.02 (0.68)	-0.03 (0.70)
**Age**				**		**
Under 30	0.02 (0.95)	-0.07 (0.64)	-0.006 (0.73)	0.23 (0.86)	0.08 (0.69)	0.24 (0.70)
30 - 49	0.03 (0.83)	0.03 (0.69)	0.02 (0.67)	-0.01 (0.81)	-0.01 (0.68)	-0.02 (0.67)
50 and older	0.12 (0.63)	-0.08 (0.72)	-0.14 (0.54)	0.21 (0.81)	0.16 (0.73)	-0.002 (0.76)
**Child Age**	***	**		***	***	
5 and Under	-0.55 (0.65)	-0.20 (0.53)	-0.13 (0.61)	-0.24 (0.87)	-0.21 (0.65)	-0.07 (0.70)
6 - 13	0.13 (0.80)	0.06 (0.68)	0.04 (0.66)	0.06 (0.84)	0.02 (0.67)	0.04 (0.66)
13 - 17	0.13 (0.84)	-0.02 (0.76)	-0.02 (0.71)	0.17 (0.67)	0.14 (0.70)	0.02 (0.64)
18 and Over	0.31 (0.80)	0.33 (0.63)	0.08 (0.73)	0.24 (0.91)	0.22 (0.79)	-0.01 (0.74)
**Race**					*	**
Asian	0.06 (0.86)	0.01 (0.58)	-0.20 (0.48)	0.08 (0.93)	-0.18 (0.69)	-0.20 (0.60)
Black	0.003 (0.94)	-0.10 (0.79)	-0.19 (0.78)	-0.02 (1.07)	-0.27 (0.71)	-0.36 (0.75)
Hispanic	0.12 (0.82)	-0.03 (0.71)	0.08 (0.64)	-0.14 (0.86)	0.11 (0.86)	-0.13 (0.89)
Other	0.38 (1.15)	0.18 (0.76)	-0.01 (0.80)	0.31 (0.96)	0.12 (0.57)	0.07 (0.62)
White	-0.0006 (0.80)	0.02 (0.67)	0.01 (0.66)	0.03 (0.80)	0.03 (0.68)	0.04 (0.67)
**School Closed**	***					
School Closed but Classed Resumed Online	0.13 (0.81)	0.01 (0.68)	0.02 (0.66)	0.07 (0.80)	-0.02 (0.67)	-0.05 (0.67)
School Closed but Classes Did Not Resume	-0.09 (0.85)	0.05 (0.73)	0.05 (0.67)	-0.004 (0.85)	0.01 (0.70)	0.04 (0.68)
School Did Not Close	-0.18 (0.92)	-0.07 (0.60)	-0.31 (0.56)	-0.01 (0.69)	0.07 (0.49)	0.06 (0.65)
Not Applicable	-0.32 (0.79)	0.06 (0.70)	-0.09 (0.71)	0.18 (1.02)	0.31 (0.84)	0.12 (0.86)
**Essential worker in family**						
No	0.09 (0.83)	0.03 (0.70)	0.01 (0.67)	0.02 (0.84)	0.01 (0.68)	-0.01 (0.68)
Yes	-0.05 (0.84)	-0.05 (0.66)	-0.03 (0.66)	0.04 (0.80)	0.02 (0.69)	0.05 (0.71)
Yes, works in COVID facility	-0.05 (0.84)	0.14 (0.69)	0.05 (0.69)	0.07 (0.92)	-0.05 (0.66)	-0.05 (0.66)
**Any family impact**	***	***	***	*		***
No	-0.03 (0.80)	-0.03 (0.66)	-0.07 (0.64)	-0.02 (0.84)	-0.01 (0.68)	-0.05 (0.66)
Yes	0.22 (0.90)	0.13 (0.74)	0.22 (0.71)	0.13 (0.77)	0.05 (0.70)	0.12 (0.71)
**Family member diagnosed**				*		*
No	0.03 (0.82)	0.01 (0.69)	-0.001 (0.66)	0.02 (0.83)	0.01 (0.68)	-0.003 (0.68)
Yes	0.24 (1.15)	0.29 (0.79)	0.23 (0.83)	0.32 (0.74)	0.08 (0.79)	0.20 (0.69)
**2 Week COVID Exposure**	***	***	***			
None	0.01 (0.81)	-0.01 (0.68)	-0.02 (0.65)	0.03 (0.83)	0.001 (0.69)	0.01 (0.67)
Exposure to person with symptoms	0.39 (1.18)	0.40 (0.63)	0.36 (0.81)	0.05 (0.80)	0.09 (0.67)	-0.04 (0.80)
Exposure to person with diagnosis	0.90 (0.80)	0.67 (0.46)	0.69 (0.66)	0.07 (0.75)	0.07 (0.72)	0.18 (0.61)
**2 Week symptom count**	***	***	***			**
None	-0.03 (0.81)	-0.04 (0.68)	-0.06 (0.65)	0.01 (0.82)	-0.01 (0.68)	-0.02 (0.67)
One	0.34 (0.80)	0.30 (0.66)	0.25 (0.63)	0.10 (0.79)	0.09 (0.69)	0.11 (0.65)
Two	0.67 (0.81)	0.31 (0.80)	0.62 (0.55)	0.03 (0.85)	-0.05 (0.60)	-0.09 (0.69)
Three or more	0.36 (1.14)	0.40 (0.54)	0.42 (0.73)	0.34 (0.89)	0.22 (0.76)	0.40 (0.82)

* $p < .05$
** $p < .01$
*** $p < .001$

S Table 2: Parent report overall mean and SD of factor scores (COVID Worries and Mood States) followed by mean and SD by demographic group and COVID-related characteristics. Significant ANOVA demographic group differences are represented by asterisks; * $p < .05$, ** $p < .01$, ***$p < .001$.

medRxiv preprint doi: https://doi.org/10.1101/2020.08.24.20181123.this version posted August 27, 2020. The copyright holder for this preprint (which was not certified by peer review) is the author/funder, who has granted medRxiv a license to display the preprint in perpetuity.
It is made available under a CC-BY 4.0 International license .

			Adult US			Adult UK			Parent US			Parent UK			
			Prior Habits Subtype			Prior Habits Subtype			Prior Habits Subtype			Prior Habits Subtype			
			1	2	3	1	2	3	1	2	3	1	2	3	4
Factor Scores	COVID Worries	p-value (η^2)	p > 0.05 (0.02)			p < 0.01 (0.01)			p < 0.01 (0.01)			p < 0.0001 (0.03)			
		Mean SD	0.02 (0.86)	-0.06 (0.85)	0.05 (0.87)	0.13 (0.87)	-0.01 (0.82)	-0.11 (0.79)	0.22 (0.82)	-0.13 (0.77)	0.01 (0.82)	0.21 (0.81)	-0.13 (0.76)	0.01 (0.81)	-0.11 (0.85)
	Prior Mood States	p-value (η^2)	p < 0.0001 (0.02)			p < 0.0001			p > 0.05 (0.004)			p < 0.0001 (0.06)			
		Mean SD	0.13 (0.66)	-0.05 (0.64)	-0.09 (0.59)	0.05 (0.65)	0.11 (0.68)	0.14 (0.62)	0.01 (0.74)	0.05 (0.64)	-0.06 (0.64)	0.24 (0.68)	-0.02 (0.63)	-0.10 (0.71)	-0.17 (0.63)
	Current Mood States	p-value (η^2)	P < 0.0001 (0.17)			p > 0.05 (0.02)			p > 0.05 (0.002)			p > 0.05 (0.008)			
		Mean SD	-0.01 (0.72)	-0.04 (0.73)	0.05 (0.73)	0.05 (0.72)	0.05 (0.76)	-0.07 (0.70)	0.01 (0.69)	-0.05 (0.66)	0.04 (0.67)	0.09 (0.68)	-0.05 (0.67)	-0.03 (0.68)	-0.04 (0.71)
Background	Sex	p-value (η^2)	p > 0.05 (0.001)			p < 0.05 (0.005)			p > 0.05 (0.01)			p > 0.05 (0.008)			
		Male	40.82%	43.54%	44.13%	36.40%	47.66%	44.16%	57.35%	53.45%	47.92%	54.61%	50.76%	46.70%	54.96%
		Female	58.23%	56.46%	54.29%	63.60%	52.34%	55.52%	41.94%	45.98%	57.89%	45.05%	49.24%	52.75%	45.04%
	Age	p-value (η^2)	p < 001 (0.04)			p < 0.0001			p > 0.05 (0.02)			p < 0.05 (0.03)			
		Under 30 %	50.63%	42.44%	34.60%	19.74%	41.87%	28.08%	11.47%	16.67%	9.90%	12.97%	5.30%	14.29%	6.61%
		30 – 49 %	28.48%	43.81%	43.81%	55.70%	34.71%	41.32%	81.00%	79.89%	83.85%	79.18%	87.88%	78.57%	87.60%
		Over 50 %	20.89%	24.72%	21.59%	24.56%	23.43%	30.60%	7.53%	3.45%	6.25%	7.85%	6.82%	7.14%	5.79%
	Child Age	p-value (η^2)	**			**			p < 0.0001 (0.40)			p < 0.0001 (0.40)			
		5 and Under %	**	**	**	**	**	**	5.04%	24.14%	18.23%	5.80%	21.37%	7.69%	40.50%
		6 – 13 %	**	**	**	**	**	**	50.72%	66.09%	72.92%	46.76%	73.28%	67.58%	54.96%
		14 – 17 %	**	**	**	**	**	**	37.77%	9.77%	8.85%	41.64%	5.34%	21.43%	4.13%
		18 and Older %	**	**	**	**	**	**	6.47%	**	**	5.80%	**	3.30%	0.41%
	Race	p-value (η^2)	p < 0.01 (0.04)			p > 0.05 (0.02)			p > 0.05 (0.03)			p > 0.05 (0.01)			
		Asian %	16.46%	17.34%	8.57%	4.82%	8.54%	6.94%	1.08%	4.60%	3.12%	5.12%	4.55%	4.95%	3.72%
		Black %	8.54%	8.12%	8.25%	5.26%	4.68%	3.47%	7.89%	4.02%	3.65%	5.80%	5.30%	4.40%	3.31%
		Hispanic %	20.25%	14.39%	13.95%	0.88%	2.75%	1.58%	15.77%	12.07%	15.10%	1.71%	0.76%	2.20%	1.65%
		Other %	4.75%	5.90%	6.03%	3.95%	6.06%	3.79%	5.73%	4.02%	4.17%	3.41%	1.52%	2.20%	3.31%
		White %	50.00%	54.24%	63.49%	85.09%	77.96%	84.23%	69.53%	75.29%	73.96%	83.96%	87.88%	85.26%	88.02%
	Household Density	p-value (η^2)	p < 0.01 (0.01)			p > 0.05 (0.03)			p > 0.05 (0.005)			p > 0.05 (0.03)			
		Mean (SD)	5.06 (3.05)	5.53 (3.32)	5.76 (3.02)	5.96 (2.48)	5.59 (2.79)	6.27 (2.78)	5.90 (2.96)	6.56 (3.25)	6.76 (3.39)	5.86 (2.47)	6.60 (2.59)	5.60 (2.55)	6.48 (2.39)
	Rooms in House	p-value (η^2)	p < 0.01 (0.009)			p > 0.05 (0.03)			p < 0.01 (0.01)			p > 0.05 (0.04)			
		Mean (SD)	0.91 (0.56)	0.71 (0.51)	0.63 (0.46)	0.69 (0.56)	0.71 (0.51)	0.63 (0.46)	0.89 (0.53)	0.82 (0.51)	0.81 (0.52)	0.81 (0.53)	0.70 (0.37)	0.88 (0.50)	0.71 (0.47)
COVID Impact	School Closed	p-value (η^2)	p > 0.05 (0.02)			p < 0.01 (0.03)			p < 0.001 (0.06)			p < 0.01 (0.05)			
		School Closed but Classes Resumed Online %	26.43%	24.62%	19.75%	6.28%	15.86%	8.09%	74.64%	60.92%	69.93%	47.72%	46.97%	44.51%	32.64%
		School Closed but Classes Did Not Close %	0.64%	0.76%	1.59%	3.14%	3.68%	4.21%	16.30%	19.54%	24.61%	45.36%	45.36%	49.54%	57.44%
		School Did Not Close %	0.96%	1.89%	3.82%	2.24%	3.12%	3.24%	2.90%	4.60%	1.05%	3.09%	7.58%	2.75%	7.44%
		Not Applicable %	71.97%	72.73%	74.84%	88.34%	77.24%	84.47%	6.16%	14.94%	4.71%	4.12%	3.03%	3.30%	2.48%
	Job Loss	p-value (η^2)	p < 0.01 (0.02)			p < 0.05 (0.02)			**			**			
		Job Prior to Pandemic and Still Working %	40.58%	44.36%	48.09%	53.54%	42.58%	25.24%	**	**	**	**	**	**	**
		Job Prior to Pandemic and Not Still Working %	24.60%	27.82%	29.30%	24.34%	24.65%	51.12%	**	**	**	**	**	**	**
		Did Not Have Job Prior to Pandemic %	34.82%	27.82%	22.61%	22.12%	32.77%	23.64%	**	**	**	**	**	**	**

Subtype 1 Subtype 2 Subtype 3 * p < .05 ** p < .01 *** p < .001

S Table 3. Prior Habits subtypes are indicated by color (Subtype 1, purple; Subtype 2, blue; Subtype 3, orange). Significant ANOVA group differences (COVID Worries, Prior Mood States, and Current Mood States) and Chi-Square group differences (Sex, Age, Child Age, Race, School Closed, and Job Loss) are represented by white asterisks; * *p* < .05, ** *p* < .01, ****p* < .001 and by color according which subtype significant differences were observed.

medRxiv preprint doi: https://doi.org/10.1101/2020.08.24.20181123.this version posted August 27, 2020. The copyright holder for this preprint (which was not certified by peer review) is the author/funder, who has granted medRxiv a license to display the preprint in perpetuity.
It is made available under a CC-BY 4.0 International license .

Life Changes ICC		Behavior & Media ICC			Substance Use ICC		
	Current Mean (SD)		Prior Mean (SD)	Current Mean (SD)		Prior Mean (SD)	Current Mean (SD)
**Positive Change**	0.66 (0.07)	**Bedtime Weekdays**	0.78 (0.06)	0.86 (0.08)	**Alcohol**	0.83 (0.19)	0.66 (0.32)
**Time Outside**	0.66 (0.08)	**Bedtime Weekends**	0.80 (0.07)	0.86 (0.04)	**Vaping**	0.92 (0.11)	0.98 (0.02)
**Contacts Changed**	0.36 (0.12)	**Hours of Sleep Weekdays**	0.80 (0.05)	0.79 (0.08)	**Tobacco**	0.99 (0.01)	0.98 (0.07)
**Family Change**	0.58 (0.06)	**Hours of Sleep Weekends**	0.78 (0.04)	0.77 (0.09)	**Marijuana**	0.98 (0.03)	0.95 (0.05)
**Friends Change**	0.70 (0.13)	**Exercise**	0.76 (0.05)	0.82 (0.07)	**Opiates**	0.80 (0.36)	0.78 (0.37)
**Cancelations Difficulty**	0.64 (0.12)	**Time Outdoors**	0.75 (0.04)	0.82 (0.06)	**Other Substances**	0.95 (0.05)	0.92 (0.09)
**Living Difficulty**	0.65 (0.12)	**TV & Media**	0.62 (0.14)	0.63 (0.12)	**Sleeping Meds**	0.86 (0.16)	0.82 (0.12)
**In-Person Conversation**	0.84 (0.21)	**Social Media**	0.82 (0.05)	0.85 (0.04)			
**Restriction Stress**	0.68 (0.08)	**Video Game**	0.84 (0.04)	0.86 (0.03)			
**Distancing Difficulty**	0.48 (0.13)						
**Family Change Stress**	0.60 (0.09)						
**Friends Change Stress**	0.62 (0.08)						
**Financial Difficulty**	0.78 (0.06)						

S Table 4. Intraclass Correlation Coefficient (ICC) mean and standard deviation for Behavior & Media, Life Changes, and Substance Use variables.

medRxiv preprint doi: https://doi.org/10.1101/2020.08.24.20181123.this version posted August 27, 2020. The copyright holder for this preprint
(which was not certified by peer review) is the author/funder, who has granted medRxiv a license to display the preprint in perpetuity.
It is made available under a CC-BY 4.0 International license .

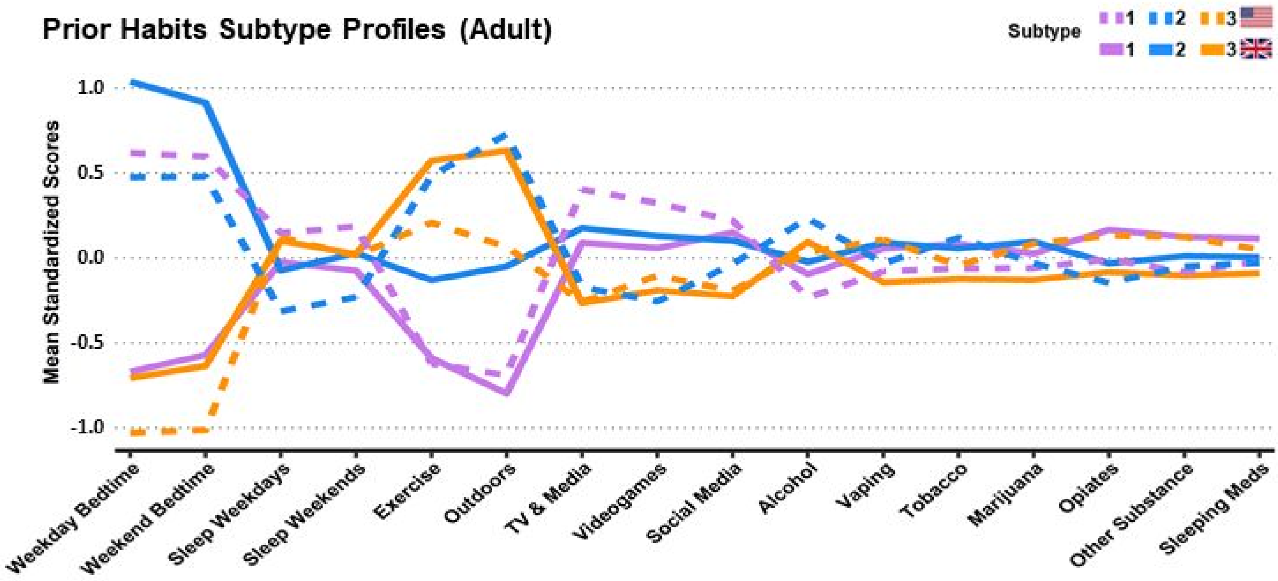

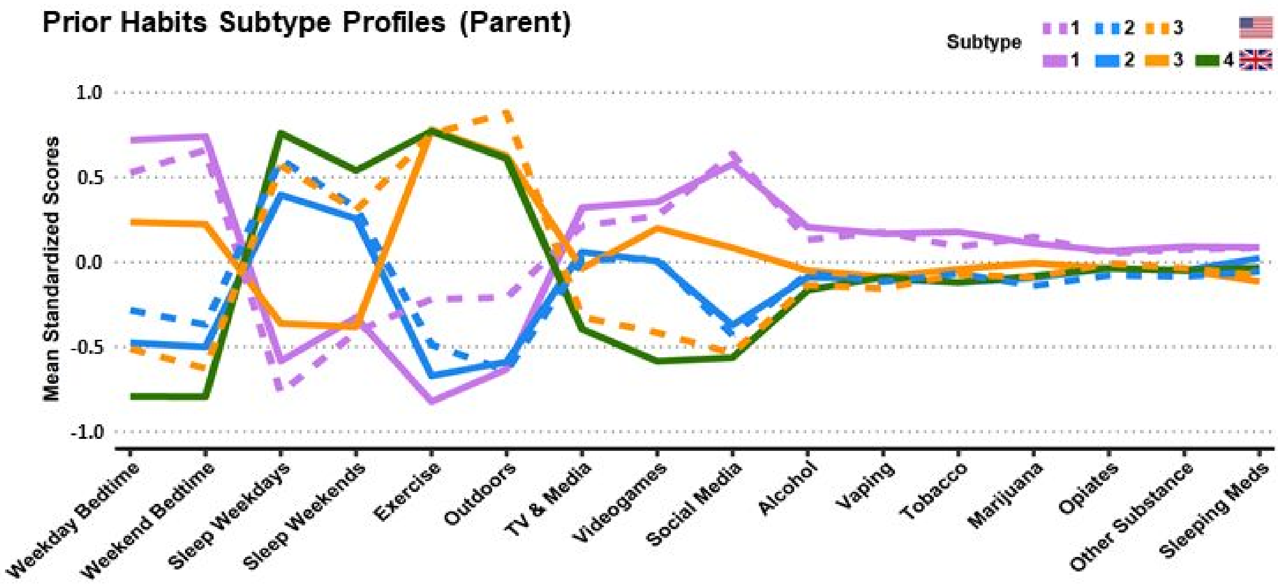

S. Figure 3: Prior Habit Subtype profiles from adult self-reports and parent reports. Mean normalized profile loadings are displayed on the y-axis. US subtypes in solid lines, UK in dashed lines. Notes: Δ Family Relations and Δ Friends Relationships were reverse coded to facilitate Subtype interpretation; higher scores indicate worsening quality of the relationships. Prior to the community detection analyses In-Person Conversation was re-coded into tertiles.

medRxiv preprint doi: https://doi.org/10.1101/2020.08.24.20181123.this version posted August 27, 2020. The copyright holder for this preprint (which was not certified by peer review) is the author/funder, who has granted medRxiv a license to display the preprint in perpetuity.
It is made available under a CC-BY 4.0 International license .

	US		UK	
	In Sample	Out of Sample	In Sample	Out of Sample
	Adult Report			
COVID Worries (Correlation)	p <.00001, 0.46	p <.00001, 0.46	p <.00001, 0.51	p <.00001, 0.50
Prior Mood States (Correlation)	p < .00001, 0.57	p <.00001, 0.58	p < .00001, 0.55	p <.00001, 0.54
Lifestyle Changes Subtypes	p < .00001	p < .00001	p < .00001	p < .00001
Age	p < .00001	p < .00001	p < .00001	p < .00001
	Parent Report			
COVID Worries (Correlation)	p <.00001, 0.40	p < .0005, 0.44	p <.00001, 0.46	p <.00001, 0.47
Prior Mood States (Correlation)	p <.00001, 0.64	p <.00001, 0.61	p <.00001, 0.51	p <.00001, 0.53
Lifestyle Changes Subtypes	p < .00001	p < .00001	p < .00001	p < .00001
Physical Health	p < .00001	p < .00001	p < .00001	p < .00001

S. Table 5. Random Forest tests identified the above five variables (COVID Worries, Prior Mood States, Life Changes Subtypes, Age, and Physical Health) to be the most important for predicting Current Mood States. The relationship between COVID Worries, Prior Mood States and Current Mood States was tested with Pearson correlations in and out of sample. ANOVA was used to test the difference in Current Mood States with Age (Adult Report) and parent-rated Physical Health (Parent Report) in out of sample. P values < .05 indicate significant differences in membership between Prior Habits and Life Changes subtypes. Correlation values are provided next the p value for COVID Worries and Prior Mood States.

	Adult US	Adult UK	Parent US	Parent UK
Subtype Homogeneity	p < .00001	p < .00001	p < .00001	p < .005

S. Table 6. Chi-Square tests were conducted between the Prior Habits and Life Changes subtypes to assess the existence of a differential makeup. P values < .05 indicate significant differences in membership between Prior Habits and Life Changes subtypes.
